# Using empirical dynamic modeling to identify the impact of meteorological factors on hemorrhagic fever with renal syndrome in Weifang, Northeastern China, from 2011 to 2020

**DOI:** 10.1371/journal.pntd.0012151

**Published:** 2024-06-06

**Authors:** Liang Zheng, Qi Gao, Shengnan Yu, Yijin Chen, Yuan Shi, Minghao Sun, Ying Liu, Zhiqiang Wang, Xiujun Li

**Affiliations:** 1 Department of Biostatistics, School of Public Health, Cheeloo College of Medicine, Shandong University, Jinan, Shandong, China; 2 School of International Business, Xiamen University Tan Kah Kee College, Zhangzhou, Fujian, China; 3 Institute of Infectious Disease Control and Prevention, Shandong Center for Disease Control and Prevention, Jinan, Shandong, China; Fundacao Oswaldo Cruz, BRAZIL

## Abstract

**Background:**

Hemorrhagic Fever with Renal Syndrome (HFRS) continues to pose a significant public health threat to the well-being of the population. Given that the spread of HFRS is susceptible to meteorological factors, we aim to probe into the meteorological drivers of HFRS. Thus, novel techniques that can discern time-delayed non-linear relationships from nonlinear dynamical systems are compulsory.

**Methods:**

We analyze the epidemiological features of HFRS in Weifang City, 2011–2020, via the employment of the Empirical Dynamic Modeling (EDM) method. Our analysis delves into the intricate web of time-delayed non-linear associations between meteorological factors and HFRS. Additionally, we investigate the repercussions of minor perturbations in meteorological variables on future HFRS incidence.

**Results:**

A total of 2515 HFRS cases were reported in Weifang from 2011 to 2020. The number of cases per week was 4.81, and the average weekly incidence was 0.52 per 1,000,000. The propagation of HFRS is significantly impacted by the mean weekly temperature, relative humidity, cumulative rainfall, and wind speed, and the ρCCM converges to 0.55,0.48,0.38 and 0.39, respectively.

The graphical representation of the relationship between temperature (lagged by 2 weeks) and the incidence of HFRS exhibits an inverted U-shaped curve, whereby the incidence of HFRS culminates as the temperature reaches 10 °C. Moreover, temperature, relative humidity, cumulative rainfall, and wind speed exhibit a positive correlation with HFRS incidence, with a time lag of 4–6 months.

**Conclusions:**

Our discoveries suggest that meteorological factors can drive the transmission of HFRS both at a macroscopic and microscopic scale. Prospective alterations in meteorological conditions, for instance, elevations in temperature, relative humidity, and precipitation will instigate an upsurge in the incidence of HFRS after 4–6 months, and thus, timely public health measures should be taken to mitigate these changes.

## Author summary

Hemorrhagic Fever with Renal Syndrome (HFRS) is a serious disease caused by the Hantavirus and transmitted by rodents. Meteorological factors, such as temperature, humidity, precipitation, and wind speed, can influence the occurrence, spread, and outbreak of HFRS. However, the relationship between climate change and its impact on the disease is complex, and more research is needed to provide a strong scientific basis for the development of adaptation strategies. This study employs a novel technique called Empirical Dynamic Modeling (EDM) to investigate the time-delayed non-linear relationships between meteorological factors and HFRS outbreaks in Weifang. The results show that meteorological factors can drive the transmission of HFRS both at a macroscopic and microscopic scale. The relationship between temperature (lagged by 2 weeks) and HFRS incidence exhibits an inverted U-shaped curve, with the highest incidence occurring at 10 °C. Changes in meteorological conditions, such as increases in temperature, relative humidity, and precipitation, can lead to an upsurge in the incidence of HFRS after 4–6 months. The findings of this study are crucial for public health officials and policymakers to take timely measures to mitigate the impact of climate change on the spread of HFRS.

## Introduction

Hemorrhagic Fever with Renal Syndrome (HFRS) is a rodent-borne disease of serious human health risk caused by the Hantavirus of the Bunyaviridae family [1]. Main clinical manifestations include fever, vomiting, abdominal pains, headache, hypotension, acute kidney damage, thrombocytopenia, and shock [2]. Currently, there are multiple views on the transmission routes of HFRS, and the main transmission routes include three categories: animal-borne transmission (including wound, respiratory, and gastrointestinal transmission), insect-borne transmission (mite transmission), and vertical transmission [3,4].

The epidemiological characteristics of HFRS are affected by various factors, including meteorological factors, rodent density, urbanization level, and vaccination [5–8]. Meteorological factors influence the incidence of HFRS by affecting infection rates and population dynamics of hosts, the regeneration of mites, and the contact rate between rodents and human beings [9–11]. According to previous studies, climate factors such as temperature, humidity, precipitation, and wind speed have substantial impacts on the occurrence, spread, and outbreak of HFRS [12,13]. However, due to the complexity of the relationship between meteorological factors climate, and impact mechanisms and the heterogeneity of some research findings, more relevant research is needed in the future to provide a strong scientific basis for the development of adaptation strategies.

Conventional statistical methods, such as the Generalized Additive Model (GAM), Distributed Lag Nonlinear Model (DLNM), and Seasonal Autoregressive Integrated Moving Average with exogenous variables (SARIMAX) models, have been employed to investigate the relationship between meteorological factors and HFRS outbreaks [14–18]. In these methods, meteorological variables that potentially affect the spread of HFRS are typically screened based on their correlation. However, correlation does not imply causality, and the presence of confounding factors may lead to a pseudo-correlation. Furthermore, nonlinearity can obscure the relationship between variables such that even when a clear effect exists, the correlation may not be observable [19]. Apparent relationships between variables can switch spontaneously in nonlinear systems as a result of mirage correlations or a threshold change in regime, and correlation can lead to incorrect and contradictory hypotheses. Nevertheless, our goal remains to determine what meteorological factors can drive the propagation of HFRS. Alves et al. [20] used a dynamic transfer function embedded in a non-Gaussian response state-space model to solve nonlinear problems, while this paper uses a new technique that can detect relationships from nonlinear dynamical systems.

In this paper, we use empirical dynamic modeling (EDM)—a mechanical, equation-free, data-driven approach that considers the contextual dependence of ecological drivers to identify and model the mechanisms driving the prevalence of HFRS [21]. Through the application of the EDM methodology to examine the HFRS and meteorological monitoring data from Weifang City, gathered during 2011–2020, this paper mainly addresses the following questions:

1. What meteorological factors are associated with outbreaks of hemorrhagic fever with renal syndrome?
2. In what ways do meteorological factors influence the spread of HFRS?

## Materials and methods

### Study area

**Fig 1:**
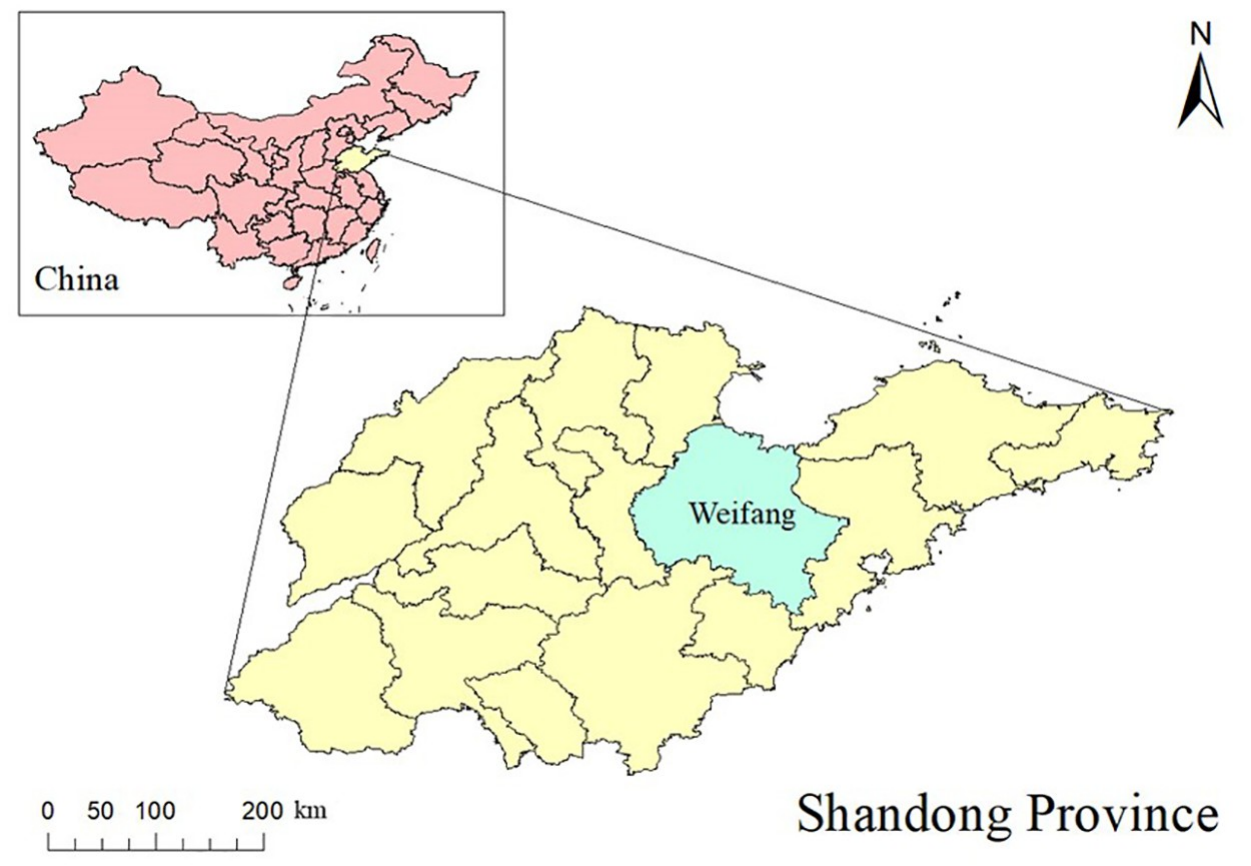
The geographical location of Weifang in China. The base map is from the data center for geographic sciences and natural sources research, CAS (https://www.resdc.cn/DOI/DOI.aspx?DOIID=121).

Weifang (Fig 1), one city of Shandong province of China, belongs to the north temperate monsoon area, back land surface sea, is located between longitudes 118 °17’E and 120 °0’E, latitudes 35 °71’N and 37 °30’N. Affected by the Eurasian continent and the Pacific Ocean, Weifang has a semi-humid continental climate with characteristics of concentrated rainfall, rain, and heat over the same period, short springs and autumns, and long summers and winters. At the end of 2022, the city has 4 districts, 2 counties, and 6 county-level cities under its jurisdiction, with a total area of 16,167.23 square kilometers and a permanent population of 9,386,705.

### Data sources

The epidemic data of HFRS from January 2011 to December 2020 were extracted from the National Notifiable Disease Surveillance System for Disease Control and Prevention. The diagnosis of HFRS was based on the clinical criteria established in the ‘Diagnostic criteria for epidemic hemorrhagic fever’ published by the National Health Commission of the People’s Republic of China (which can be found at https://icdc.chinacdc.cn/zcfgybz/bz/202112/t20211202_253342.html)[22]. Demographic information about Weifang, from 2011 to 2020, was retrieved from the Statistical Yearbook of Shandong Province. The weekly HFRS incidence (per 1,000,000) for Weifang was calculated by the epidemic-reported data and population data.

Hourly data for meteorological variables from 2011 to 2020 were obtained from ERA5, the fifth-generation ECMWF reanalysis system for global climate and weather data spanning the past four to seven decades (https://www.ecmwf.int/en/forecasts/dataset/ecmwf-reanalysis-v5) [23]. For variables like temperature, humidity, and wind speed, we computed the average of all raster data within the Weifang area. As for precipitation, we summed up all values within the defined area to obtain the total precipitation. We then calculated weekly averages of these variables, including mean temperature (°C), mean relative humidity (%), cumulative rainfall (mm), and mean wind speed (m/s).

## Empirical dynamic modeling (EDM)

EDM is a developing non-parametric framework grounded on Takens’s theorem of state-space reconstruction (SSR) for modeling nonlinear dynamic systems [21,24]. The procedure of using lagged coordinate embedding of time series data to reconstruct the attractor for an unknown system from nature is called state-space reconstruction. The dynamic attractor is a complete representation of the system, enabling the study of the system to predict and comprehend non-parametric equations of complex systems, such as host-pathogen dynamics. More information on this can be found at http://deepeco.ucsd.edu/video-animations/.

Essentially, dynamical systems can be described as the evolution of a set of states over time based on some rules governing the movement of states in a high-dimensional state space (i.e. a manifold). Motion on the manifold can be projected onto a coordinate axis, forming a time series. Takens’s theorem[25] states that the shadow version of the dynamics reconstructed by such an embedding preserves the essential features of the true dynamics (so-called topological invariance). Therefore, based on the concept of state space reconstruction, EDM can be used to study nonlinear dynamical systems.

EDM has multiple utilities in studying dynamical systems, which in this paper mainly include: determining influencing factors, forecasting, and exploring situations of external perturbations.

### EDM: Convergent cross mapping(CCM)

CCM is one of the methods of EDM, based on nonlinear state space reconstruction that can detect causality in nonlinear dynamic systems [19]. Compared to Granger causality testing, its theoretical foundation is not yet fully developed, but it fills in the gap left by Granger causality testing in inseparable and weakly coupled systems. However, drawing causal conclusions beyond simple correlation from time series data falls under the broad category of ’time series causality,’ and all methods for measuring time series causality suffer from such limitations. Conducting CCM testing is only a necessary but not sufficient condition for causality[26]. If the CCM results show that X does not influence Y, it suggests that there may indeed be no causal relationship between X and Y. However, if the CCM results indicate that X does influence Y, it could also be influenced by other confounding factors, and in reality, there may be no causal relationship between X and Y [27]. Therefore, in this study, we primarily use the CCM method to explore the nonlinear relationships between the factors under investigation.

The fundamental concept is that if variable Y has a causal effect on variable X, then causal information of variable Y should be present in X, and hence the attractor reconstructed for variable X should be able to predict the states of variable Y. The critical criterion for estimating causal associations between two variables using CCM is checking whether the cross-mapping skill ρ monotonically increases and converges with the length of time series used in cross-mapping. By observing the convergence of ρ, we can infer the presence of causal relations between two variables.

Here, we implemented an extension of CCM that addresses this issue by considering distinct lags for cross-mapping. Consequently, we can deduce whether a driving variable exerts an influence on a response variable with a time delay [28]. This "asynchrony", which embodies the temporal lag in the response, is instrumental in discriminating between bidirectional causality and discerning the order of variables in a transitive causal chain.

In this study, CCM was employed to reveal the lag-response effects of a non-linear interaction between meteorological variables and HFRS incidence. The embedding dimension (E) was determined based on the simplex projection outcomes. The time-delayed effect (Tp) was set as 0–30 weeks for detecting the time lags. Notably, the meteorological variables under investigation display seasonality, which increases the likelihood of spurious correlations. Therefore, it is imperative to distinguish driving effects from mutual seasonality. This can be addressed by developing null tests using proxy time series [29–31]. To determine the seasonal cycle, a smoothing spline was utilized with a smoothing parameter of 0.9 applied to a climate variable as a function of the day of the year. The seasonal cycle can be calculated by subtracting the seasonal anomaly from the observed value. We then randomly added the shuffled anomalies back to the seasonal cycle to get the proxy time series. If linked to a climate variable, HFRS incidence should be more accurately predicted by the real-time series of the climate variable than by the surrogate time series. To create an ensemble of null models for the seasonal surrogate test, the shuffling procedure was repeated 100 times. The value of prediction skill ρ for the original series was compared to the null models to assess whether the result is statistically significant, using a significance level of *α* = 0.05. If the cross-mapping skill converges and passes the seasonality test, then there exists a one-way association between the two tested variables.

### EDM: Scenario exploration

Scenario exploration with multivariate EDM allowed us to assess the effect of a small change in climate variables on HFRS incidence, across different states of the system [29]. Deyle et al. utilized the EDM to explore the relationship between temperature, humidity, and influenza outbreaks and found a U-shaped relationship between humidity and influenza [29]. Nova et al. employed the EDM to investigate the relationship between meteorological factors and the outbreak of dengue fever, revealing climate variables drive dengue dynamics in a nonlinear and complex, yet predictable way [30]. In this study, we employ this methodology to investigate the relationship between climate variables and HFRS. EDM can leverage historical trajectories to forecast future values by relying on the behavior of the nearest neighbors in the reconstructed state space, as close vectors in state space tend to evolve similarly over time [24,32]. To gauge the sensitivity of HFRS outbreaks to the environment, we projected the effects of a minor increase in a climate variable with different lags on HFRS incidence. For each historical time point, *t*, we predicted HFRS incidence *Y*(*t*) with a small increase (+Δ*X*) and decrease (−Δ*X*) in climate driver *X*(*t*), which was historically measured. Regarding each potential climatic factor, the disparity in dengue projections resulting from these minor adjustments can be expressed as $\Delta Y=Y\left(t+1\right)\left[X\left(t\right)+\frac{\Delta X}{2}\right]-Y\left(t+1\right)\left[X\left(t\right)-\frac{\Delta X}{2}\right]$ [30]. Here, *Y*(*t* + 1) denotes a function of *X* and all other state variables, and we employed Δ*Y*/Δ*X* to approximate the sensitivity of HFRS infection to the climate driver *X* at time *t*. Δ*X* corresponds to ∼5% of the SD of these meteorological variables. We repeated this over all time steps in our time series for the climate variables with different lags that we previously tested to obtain their relationships with HFRS incidence across different system states.

All analyses were conducted in R version 4.0.5 using the package rEDM.

## Results

### Descriptive analysis

From 2011 to 2020, a total of 2515 cases of HFRS were reported in Weifang, with an average of 4.81 cases per week and a maximum of 38 cases reported in a single week. Table 1 presents the summary statistics for the incidence of HFRS and meteorological variables in Weifang during this period. The temperature in Weifang ranged from -9 °C to 30 °C, with a mean temperature of 13.91 °C. The relative humidity ranged from 8.20% to 89.60%, with a mean of 58.93%. The total rainfall ranged from 0mm to 190mm, with a mean of 19.38mm, and the wind speed ranged from 1.48m/s to 4.68m/s, with a mean of 2.73m/s.

**Table 1:** Summary statistics of weekly HFRS incidence and meteorological factors in Weifang, China, 2011–2020.

Variable	Mean	S.D.	min	25th	Median	75th	Max
Incidence of HFRS(per 1,000,000)	0.52	0.48	0.00	0.21	0.75	4.00	4.12
Temperature (°C)	13.91	10.27	-9.00	3.75	15.49	23.73	30.13
Relative Humidity (%)	58.93	16.43	1.97	48.62	60.51	71.14	89.57
Rainfall (mm)	19.38	30.31	0.00	1.11	7.42	24.31	190.60
Wind Speed(m/s)	2.73	0.56	1.49	2.31	2.68	3.15	4.69

**Fig 2:**
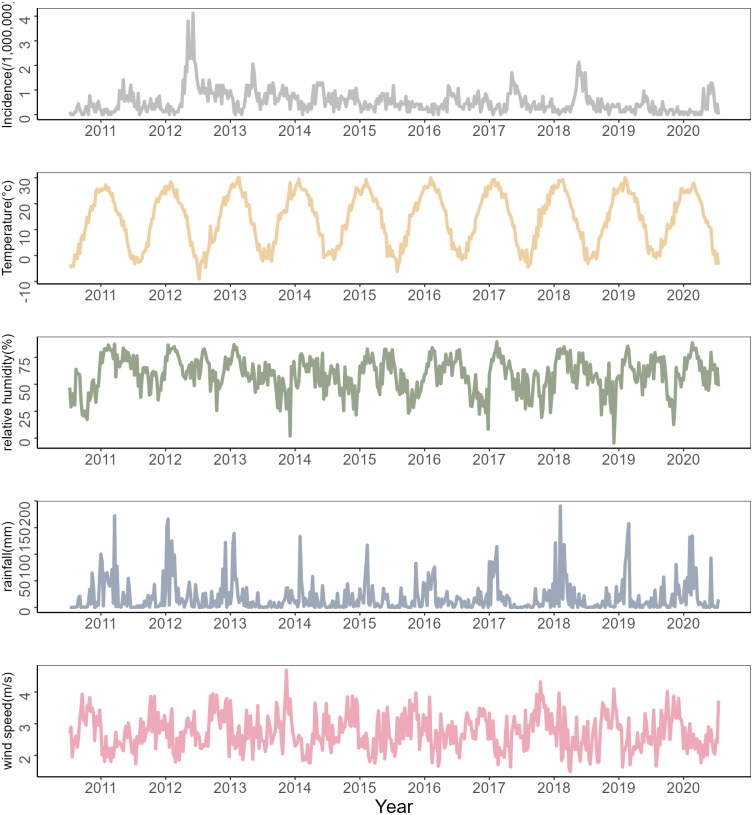
Time series of the HFRS incidence and meteorological factors. Time series (2011–2020) of weekly HFRS incidence(per 1,000,000), weekly average temperature (°C), weekly average relative humidity (%), total weekly rainfall (mm), and weekly average wind speed (m/s).

**Fig 3:**
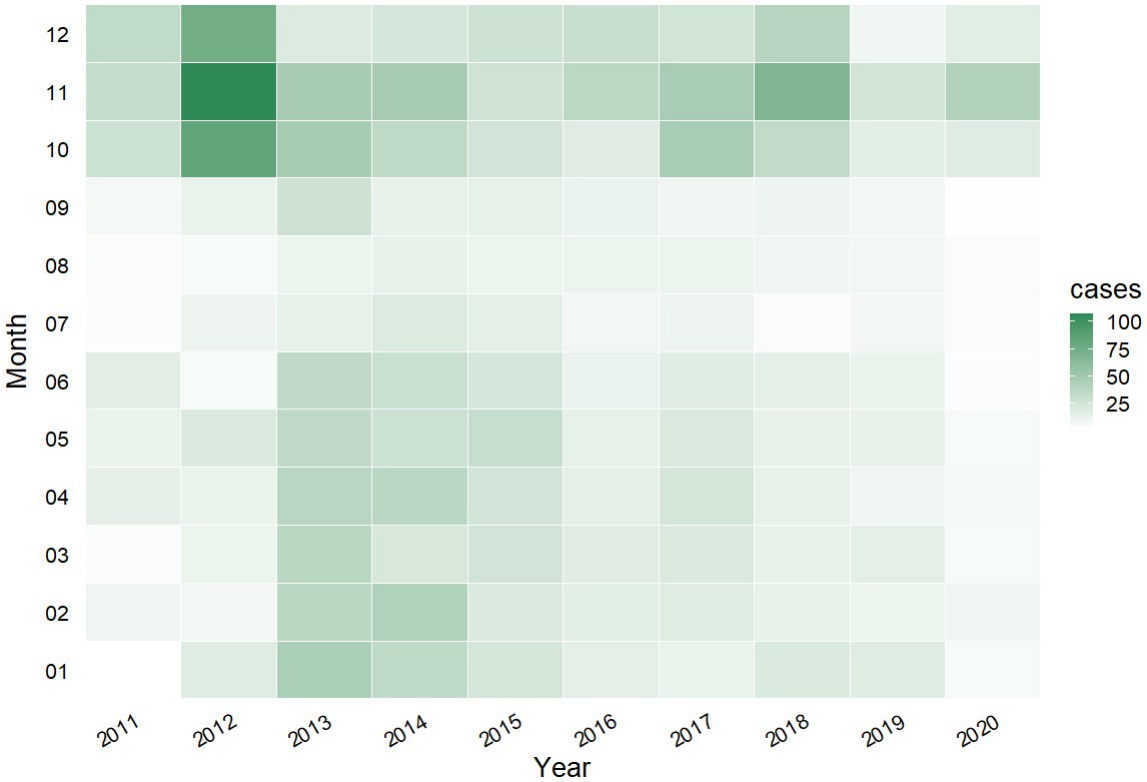
Monthly distribution of HFRS cases (2011–2020, Weifang).

The time series of HFRS cases and meteorological factors are shown in Fig 2. All climate variables demonstrate a clear seasonal cycle trend, with a wet and warm summer season followed by a colder and less wet winter season. Fig 3 further supports the existence of a seasonal pattern in HFRS case distribution, with a bimodal distribution recorded from March to June and from October to December, with the latter peak more prominent. HFRS incidence has remained relatively stable over the study period, except for a significant increase in 2012. The incidence of HFRS in Weifang exhibits a bimodal distribution pattern, characterized by two distinct peaks observed from March to June and from October to December, respectively, with the latter exhibiting a more salient prominence.

### Climate drivers of HFRS dynamics with different lags

**Fig 4:**
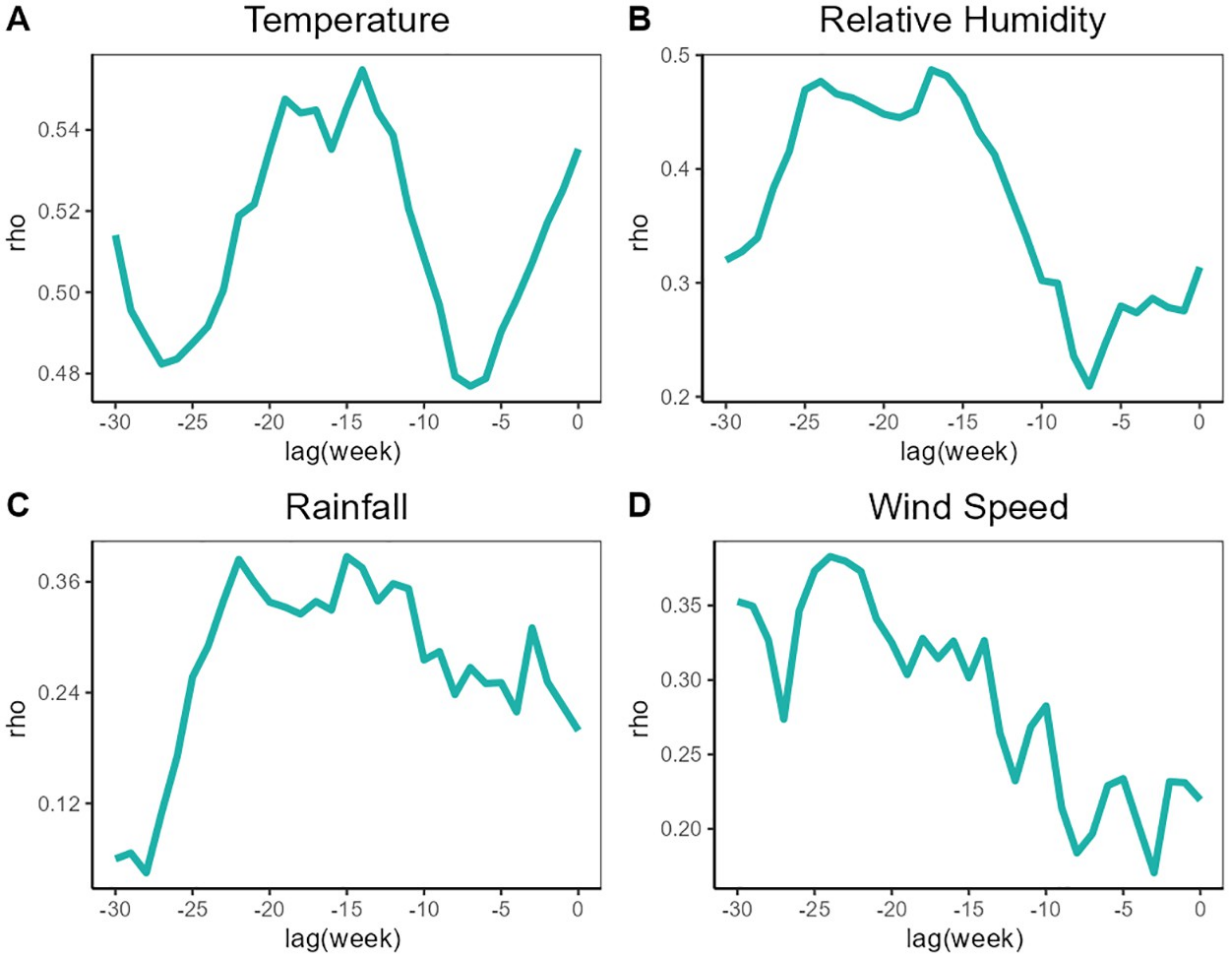
The cross-map skill (ρCCM) of meteorological variables with HFRS incidence at different lags. Based on cross-correlation with HFRS incidence, the temperature has the optimal correlation at a 14-week lag (A), relative humidity at a 16-week lag (B), rainfall at a 15-week lag (C), and wind speed at a 24-week lag (D).

**Fig 5:**
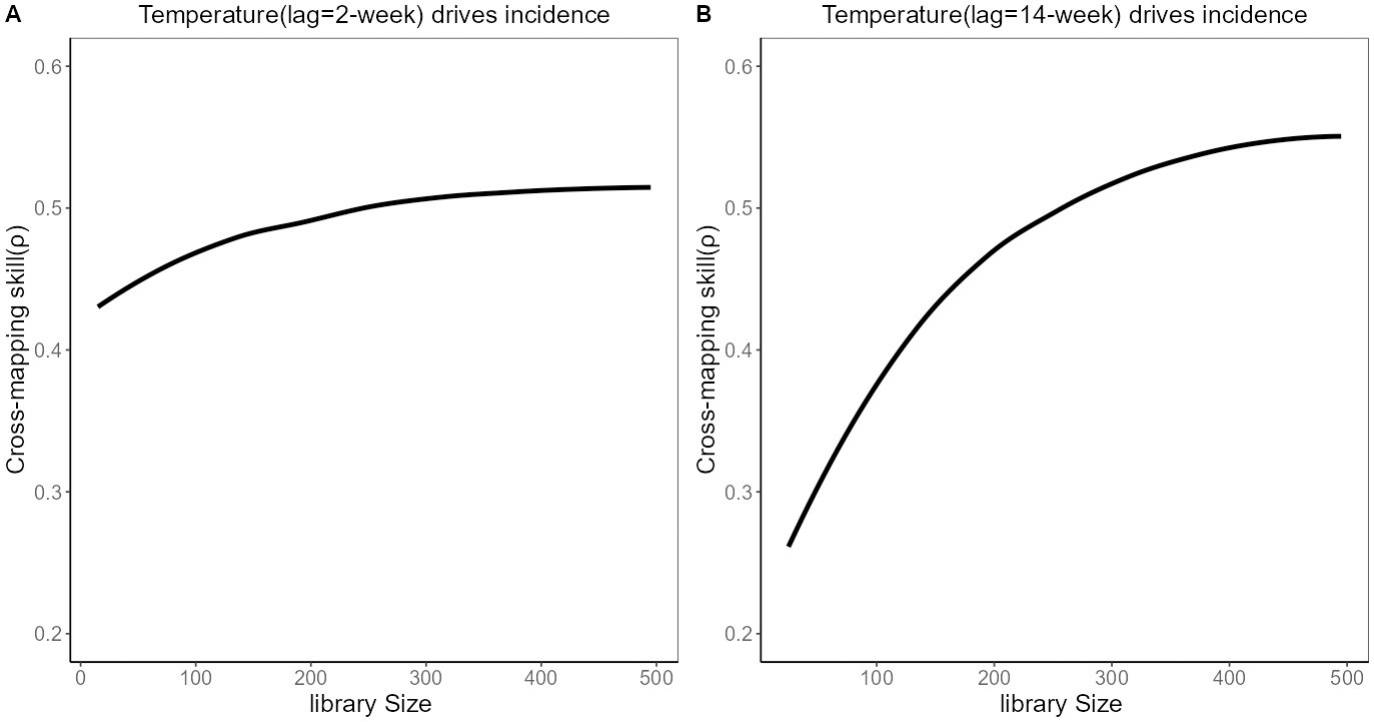
Cross-mapping between HFRS incidence and temperature with different lags. The cross-mapping skill ρ between temperature with a lag of 2 weeks and the HFRS incidence increases with the library size(the sample size) and finally converges to 0.52 (A). The cross-mapping skill ρ between temperature with a lag of 14 weeks and the HFRS incidence increases with the library size and finally converges to 0.55 (B). Temperature with different lags can drive the incidence of HFRS.

We used CCM to explore whether meteorological variables with different lag periods can drive HFRS incidence. The process of determining the optimal embedding dimension and the detection of evidence for nonlinear dynamics is depicted in S1 and S2 Figs in Supporting information(The definitions of EDM related nouns are in S1 Text). We conducted a grid search for lag stages of climatic variables within the range of 0–30 weeks. The determination of this time range was based on reference literature exploring the relationship between meteorological factors and HFRS [14]. In Fig 4, we showed cross-map skill (ρCCM) of meteorological factors with a maximum lag of 30 weeks on the incidence of HFRS. In Fig 5, the detailed CCM results between temperature and HFRS across various lag periods, as depicted in Fig 4, are presented. The detailed CCM results for other variables are included in S3, S4 and S5 Figs. The ρCCM analysis revealed dual peaks for the relationship between temperature and HFRS incidence, with the first peak observed at a lag of 2 weeks and the second peak at a lag of 14 weeks. Relative humidity was linked to HFRS incidence after a lag of 16–24 weeks, while total rainfall was linked to HFRS incidence after a lag of 12–23 weeks. Wind speed also exhibited a link with HFRS incidence, with a lag of 23–25 weeks. According to prior research, the incubation period of HFRS ranges from 2 to 4 weeks [33]. To investigate the effects of meteorological variables at different time scales on HFRS, we picked the time lags with maximum ρ (Based on cross-correlation with HFRS incidence, the temperature has the optimal correlation at a 2-week lag and 14-week lag, relative humidity at a 16-week lag, rainfall at a 15-week lag, wind speed at a 24-week lag), and 2-week lag (Mean incubation period of HFRS) to conduct the following tests.

**Fig 6:**
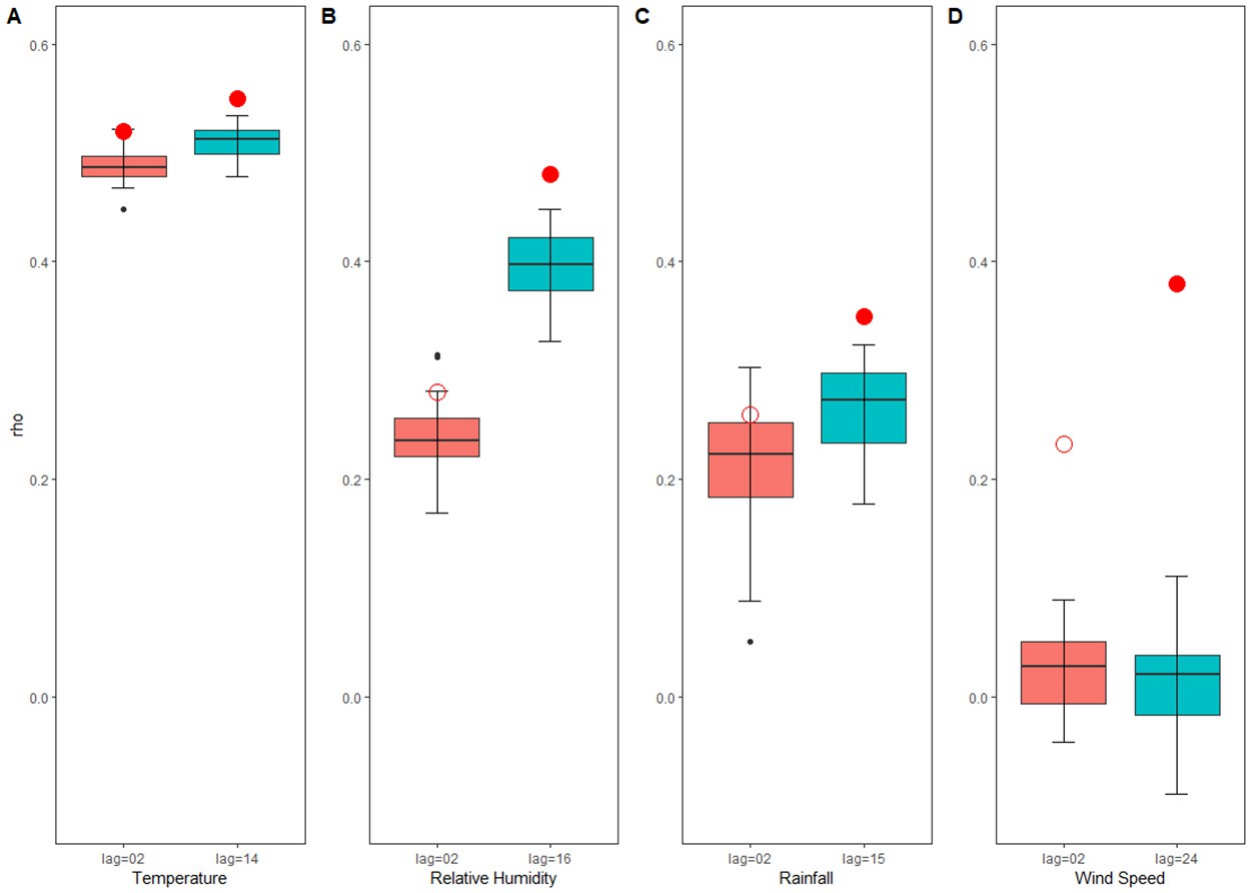
Detecting cross-map relationships beyond shared seasonality of meteorological variables on HFRS. The box-and-whisker plots depict the null distributions of ρCCM that are anticipated from random surrogate time series that possess identical seasonality to the authentic environmental driver. Red circles show the cross-map skill (ρCCM) for observed HFRS incidence predicting purported seasonal drivers, and filled circles indicate that the measured ρCCM converges and is significantly better than the null distributions (*P* ≤ 0.05).

To avoid seasonal interference in the time series of meteorological factors, we built a seasonal null model and statistically tested for statistically significant differences in cross-mapping skill between the model that used the real data versus the null models. In Fig 6, the box-and-whisker plots of the null distributions for ρCCM demonstrated the cross-map relationships beyond the shared seasonality of environmental drivers on HFRS. Based on the results, we can observe that the ρCCM of temperature with the incidence of HFRS was statistically significant when the lag was 2 and 14 weeks. In contrast, the ρCCM of relative humidity, total rainfall, and wind speed with the incidence of HFRS were statistically significant when the lag was 16, 15, and 24 weeks, respectively. Thus, we can conclude that temperature, relative humidity, precipitation, and wind speed are drivers of the incidence of HFRS.

## Scenario exploration of external climate variables perturbation

**Fig 7:**
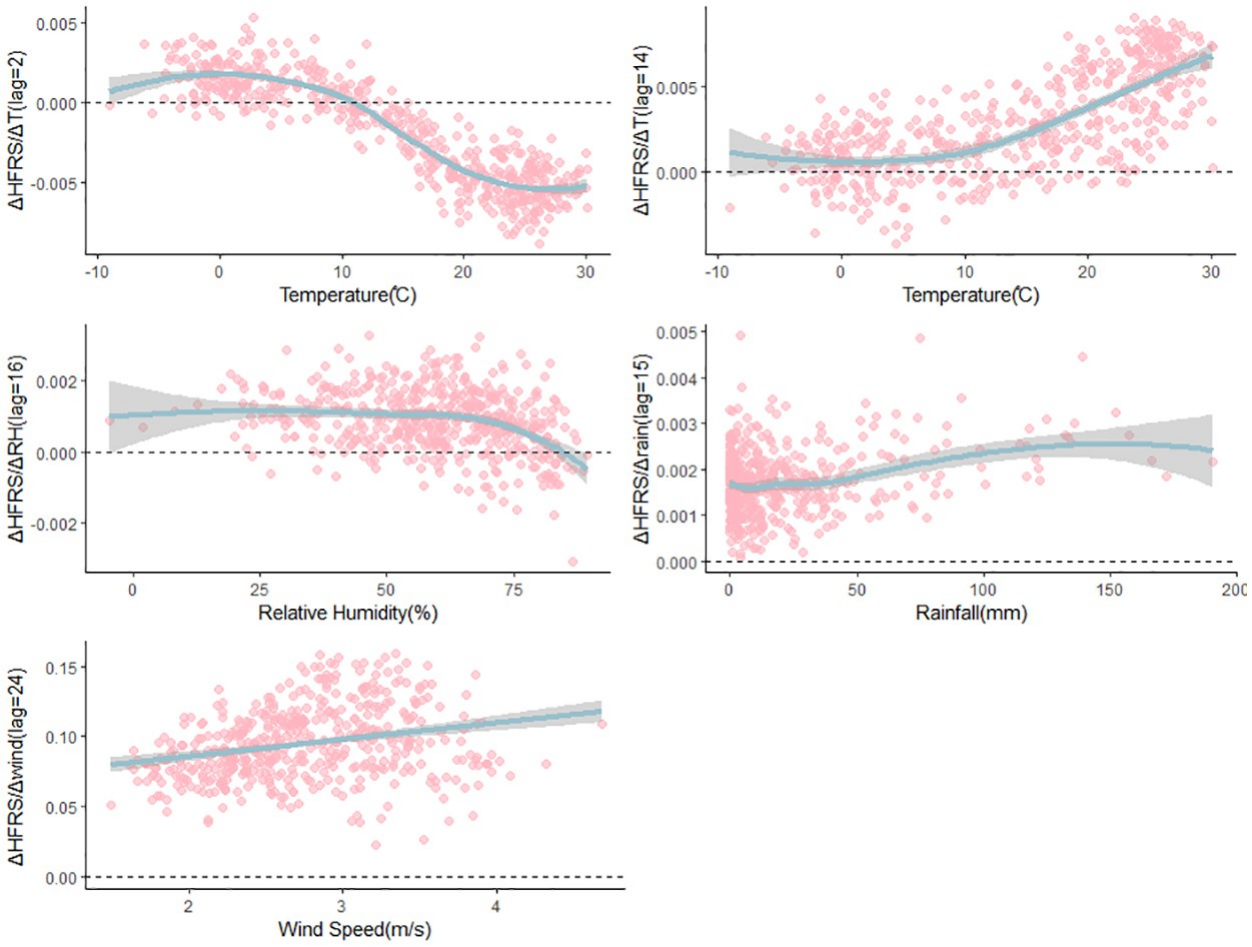
Scenario exploration with multivariate EDM. Provides an assessment of the impact of climatic factors on the incidence of HFRS by forecasting the alteration in HFRS incidence (ΔHFRS) due to minor variations in temperature (ΔT), relative humidity (ΔRH), precipitation (Δrain), and wind velocity (Δwind).

Fig 7 displays the lag-specific relationship between various climate variables and HFRS incidence. Here we predicted the hypothetical change in HFRS incidence, denoted Δ HFRS, at historical points that would occur from small increases and decreases in the meteorological factors which were validated in the previous section of the article, to explore how climate perturbations might affect the incidence of HFRS.

In general, a rise in temperature is associated with an increase in the number of cases, although the magnitude of this effect depends on both the current and lagged temperature. Fig 7B shows that the effect of temperature (14-week lag period) on HFRS incidence is always positive (ΔHFRS/ ΔT > 0). This could be attributed to the fact that the temperature conditions during the 14 weeks preceding the winter peak of HFRS may have an indirect impact on rodent reproduction by influencing the growth of vegetation. However, From Fig 7A, when the temperature drops below 10°C, we observe a positive relationship (ΔHFRS/ ΔT > 0) between temperature (with a 2-week lag) and HFRS incidence. At around 10°C, we see the peak of HFRS incidence, which then declines as temperatures continue to rise. This is consistent with the fact that high temperatures are generally not conducive to the persistence of the virus in the environment [34,35]. Furthermore, from an immunological perspective, the activity of immune cells may diminish in cold environments, consequently reducing the body’s resistance to viruses. This could also explain why an increase in temperature acts as a protective factor against HFRS.

Fig 7C illustrates that the effect of relative humidity (with a 16-week lag) on HFRS incidence is positive (ΔHFRS/ ΔRH> 0). Similarly, Fig 7D showed that the influence of rainfall (with a 15-week lag) on HFRS incidence is positively correlated (ΔHFRS/ Δrainfall> 0). The plausible explanation for the positive effect of relative humidity and rainfall (lag = 4 months) on HFRS incidence is that abundant summer precipitation and high relative humidity can foster crop growth, which can provide optimal conditions for the survival and proliferation of rodents. This relates to the abundance of food sources for rodent hosts and potentially the size of the rodent populations. Furthermore, as shown in Fig 7E, we observed that the effect of wind speed (with a 24-week lag) on HFRS incidence is consistently positive (ΔHFRS/ Δwind> 0), and increases with increasing wind speed.

## Discussion

In this study, we analyzed the prevalence and distribution characteristics of HFRS in Weifang City from 2011 to 2020 and explored the impact mechanism of climate variables on the outbreak of HFRS by using the EDM method. The findings of our study revealed that temperature, relative humidity, rainfall, and wind speed had a delayed non-linear effect on HFRS in Weifang.

Given the existence of spatial heterogeneity, we found that the lag time of the influence of meteorological factors on HFRS cases differs in different regions compared with existing studies. Sun et al found that temperature and humidity with a 15–16 week lag were closely correlated with HFRS, while precipitation does not affect HFRS in Huludao City [36]. Xiang et al. pooled the results of China’s 19 cities and showed that the lags with the largest effects for maximum temperature, precipitation, and relative humidity occurred in weeks 29, 22, and 16, respectively [14]. Wei et al. showed that average temperature with a 1-month lag was negatively associated with the incidence of HFRS in Guangzhou City [37]. The observed discrepancies imply that research efforts ought to be tailored to local conditions and that adaptable prevention and control measures should be implemented accordingly.

Utilizing the CCM technique, we observed that the HFRS incidence in Weifang was related to temperature with lags of about 2 and 14 weeks, humidity with lags between 16–24 weeks, precipitation with lags between 12–23 weeks, and wind speed with lags of about 23–25 weeks, respectively. While only temperature was found to be linked to HFRS at a 2-week lag, our analysis revealed that all meteorological variables under consideration were associated with the outbreaks of HFRS with a lag of approximately 4 months, which is consistent with prior investigations. We postulated that these short-term lags (lag = 2 weeks) may be linked to the survival of the HFRS virus in external environmental conditions and the frequency of contact between vector rodents and humans, while the long-term lags (lag = 4 months) of climatic factors may have indirect effects on HFRS outbreaks by influencing crop growth and rodent population density. The relationships between meteorological factors and HFRS incidence with different time lags can be explained on two different scales: the longer ones are related to the amount of vegetation as well as rodent population dynamics, and the shorter ones are related to in vitro virus survival.

In prior studies examining the link between temperature factors and HFRS in northeastern China, varying outcomes have been reported. Lin et al. found an inverse U-shaped relationship between temperature and HFRS incidence, with the incidence of HFRS peaking at 17 °C [38]; and Xiang et al. found that the incidence of HFRS raised with temperature [14]. The incongruent findings in the aforementioned studies could potentially be attributed to differences in the explored lag times. In our investigation, we observed that an escalation in temperature (with a lag of 4 months) can elevate the susceptibility to HFRS infection in Weifang. Notably, we discovered that the impact of temperature on HFRS cases is not only positively correlated but also amplifies as the temperature rises. Temperature with lags of 4 months can cause changes in the population dynamics of HFRS host animals by affecting the density of vegetation, the gestation period, and the sexual maturity of rodents. However, we also found an inverted U-shaped relationship between temperature with lags of 2 weeks and HFRS, which peaked at 10 °C. This result is consistent with the findings of in vitro studies of hantavirus. Data gathered from controlled laboratory experiments indicate that temperature is intricately linked to the survival of the hantavirus, with elevated temperatures posing a threat to its persistence. The hantavirus can survive for more than 10 days at room temperature and more than 18 days at +4 °C [34]. As respiratory transmission is a crucial route for the spread of HFRS, inhalation of virus-laden aerosols can result in infection [9]. At a temperature of 10 °C, the rodents exhibit heightened activity and the survival rate of the hantavirus in aerosols is optimal, thus rendering it conducive for the transmission of HFRS.

Consistent with previous studies, it was observed that precipitation and humidity, with a lag period of 4 months, exhibit affirmative impacts on the emergence of HFRS. This can be attributed to the fact that elevated humidity and precipitation foster the growth of ground vegetation, thereby furnishing an abundant food source for rodents. A humid environment was conducive to the survival or reproduction of mites [39]. In previous studies, excessive precipitation was negatively associated with the occurrence of HFRS because increased precipitation can disrupt the environment in which host animals live, and frequent rainfall, especially flooding, reduces rodent-human contact, so virus transmission is limited [40]. However, due to the relatively low annual precipitation in the Weifang area, only a positive effect of precipitation on HFRS was detected.

Limited research has been conducted on the correlation between wind speed and HFRS. Luo et al. found an increased risk of HFRS when the monthly mean wind speed was favorable [41]. In our study, we found increasing wind speed with a 24-week lag contributes to the increase in HFRS incidence. The precise mechanism underlying the impact of wind speed on HFRS remains unclear; nonetheless, it is posited that wind speed may indirectly influence HFRS incidence by modulating precipitation patterns. Given Weifang’s coastal location, it is characterized by an elevational gradient from west to east, thus attracting substantial summer monsoon rainfall from the Pacific Ocean. Additionally, wind may augment the transmission of hantavirus by accelerating the airflow.

Furthermore, there are several strengths included in our study that enhance the completeness and reliability of our findings. First, the utilization of the EDM not only offers an alternative criterion for identifying relationships between meteorological factors and the incidence of HFRS cases in Weifang, but it also enables the selection of suitable meteorological variables to be incorporated into predictive statistical or dynamical models. Previous studies on the relationship between meteorological factors and HFRS have often relied on correlation analysis to explain the seasonality of HFRS incidence. However, correlation is a limited tool for understanding relationships in nonlinear systems. This insufficiency urged us to explore alternative methods for identifying the external drivers of nonlinear dynamics. Furthermore, traditional statistical models built on linear assumptions are generally ineffective in predicting disease prevalence, as the exposure-response relationship between factors influencing disease prevalence and disease prevalence itself is often nonlinear. Compared to traditional linear statistical methods, EDM has the advantage that it does not resort to any parametric assumptions and recovers the original system dynamics patterns from time series data through lagged time coordinate embedding. Second, in contrast to previous studies that have tended to adopt conservative lag time settings, our paper expands the lag range to encompass 1–30 weeks to detect the impact of meteorological factors on HFRS at longer time scales. This decision is grounded in the understanding that while the incubation period of HFRS typically ranges from 2–4 weeks, the lag period of the effect of meteorological variables on HFRS may be extended due to their influence on rodent population density via their impact on vegetation density. Last, the relationships tested by CCM can contribute to modeling future scenarios of HFRS and other diseases’ re-emergence. We projected the trend of HFRS incidence under different future climate scenarios. The results then show the influence of climate warming and increasing climate fluctuations that future predictive models can take into account.

Despite the efforts made to minimize potential confounding factors, this study is not immune to certain limitations. Firstly, in the EDM methodology, the accuracy of CCM is limited by observational errors, process noise, and the length of the time series L. Undoubtedly, data preprocessing techniques such as detrending or removing seasonal components have the potential to mitigate the issues outlined above. However, there is a possibility of losing time series features that are crucial for CCM [26]. Therefore, further research is required to refine the data preprocessing strategies for CCM. Secondly, the spatial scope of this study was confined to a single city, which limits its representativeness given the evident spatial heterogeneity of HFRS outbreaks. Subsequent research can be conducted at the national level to obtain a more comprehensive research conclusion. Finally, it is important to note that the meteorological data utilized in this study was obtained from a reanalysis dataset of European weather stations, which may not necessarily represent the precise local weather conditions in Weifang and thus could potentially lead to limitations in the accuracy and completeness of the results.

## Conclusions

Overall, our findings provide empirical evidence that temperature, relative humidity, precipitation, and wind speed with different lag times can drive HFRS dynamics in a nonlinear but predictable way, suggesting that meteorological factors can drive the transmission of HFRS not only from the macroscopic scale(affecting the reproduction of host animal populations) but also from the microscopic scale(affecting the survival of hantavirus in the air). There is generally a lag time of 4–6 months between HFRS and climatic factors, and this window period provides valuable time for an intervention. Future changes in meteorological conditions, such as rising temperatures, relative humidity, and precipitation will cause an increase in the incidence of HFRS after four months, so appropriate public health measures should be taken to mitigate these changes to address the high prevalence of HFRS in some areas. In a rapidly changing environmental world, EDM can be used to predict ecological responses to environmental change.

## Supporting information

S1 Text Definition of EDM related terms. (DOCX)

S1 Fig Optimal embedding dimension (E). The output is a data frame with columns E and rho detailing the embedding dimension and Pearson correlation coefficient between the simplex projected forecast at Tp = 1 timestep ahead, and the observed data over the pred indices. The optimal embedding dimensions for Temperature(A), Relative humidity(B), Rainfall(C), and Wind speed(D) obtained here for further analyses are 10,10,8, and 10, respectively.
(TIF)

S2 Fig Evidence of nonlinear dynamics. The S-map test for nonlinearity confirms that all variables have nonlinear state dependence. If the optimal θ>0, the forecast given by the S-map depends on the local state of the predicted points, then the system is state-dependent. If θ = 0 then all points are weighted equally, and the system is linear. By comparing the performance of equivalent linear (θ = 0) and nonlinear (θ>0) S-map models, one can distinguish nonlinear dynamical systems from linear stochastic systems. The optimal θ of Temperature(A), Relative humidity(B), Rainfall(C), and Wind speed(D) are all greater than 0, which means the systems of these variables are all nonlinear and thus motivate the use of EDM.
(TIF)

S3 Fig Cross-mapping between HFRS incidence and relative humidity with different lags. The cross-mapping skill ρ between relative humidity with a lag of 2 weeks and the HFRS incidence did not converge as library size(the sample size) increased (A). The cross-mapping skill ρ between relative humidity with a lag of 16 weeks and the HFRS incidence increases with the library size and finally converges to 0.48 (B). Relative humidity with a lag of 16 weeks can drive the incidence of HFRS.
(TIF)

S4 Fig Cross-mapping between HFRS incidence and total rainfall with different lags. The cross-mapping skill ρ between rainfall with a lag of 2 weeks and the HFRS incidence did not converge as library size(the sample size) increased and began to decline when the library size exceeded 400 (A). The cross-mapping skill ρ between rainfall with a lag of 15 weeks and the HFRS incidence increases with the library size and finally converges to 0.38 (B). Total rainfall with a lag of 15 weeks can drive the incidence of HFRS.
(TIF)

S5 Fig Cross-mapping between HFRS incidence and wind speed with different lags. The cross-mapping skill ρ between wind speed with a lag of 2 weeks and the HFRS incidence did not converge as library size(the sample size) increased (A). The cross-mapping skill ρ between wind speed with a lag of 24 weeks and the HFRS incidence increases with the library size and finally converges to 0.39 (B). Wind speed with a lag of 24 weeks can drive the incidence of HFRS.
(TIF)
