# Supplementary material for: Using empirical dynamic modeling to identify the impact of meteorological factors on hemorrhagic fever with renal syndrome in Weifang, Northeastern China, from 2011 to 2020
Source: PLoS Negl Trop Dis. 2024 Jun 6;18(6):e0012151. doi: 10.1371/journal.pntd.0012151 (PMC11185475; doi:10.1371/journal.pntd.0012151)
Supplement: S1 Text — (DOCX) [file pntd.0012151.s001.docx]

**S1 Text Definition of EDM related terms**

**1.Optimal embedding dimension**

The reconstruction will map one-to-one to the original attractor manifold if enough lags are used (i.e. if the reconstruction has a sufficiently large embedding dimension). If the embedding dimension is too small, then reconstructed states can overlap and appear to be the same even though they correspond to different states. These “singularities” will result in poor forecast performance because the system behavior cannot be uniquely determined in the reconstruction. As a consequence, we can use prediction skills as an indicator for identifying the optimal embedding dimension. We can use the Simplex() projection nearest neighbor forecasting method to identify the Optimal embedding dimension[1]

**2.Simplex Projection**

One of the most crucial aspects of SSR (State Space Reconstruction) involves determining the embedding dimension E. Simplex projection serves as a predictive method used to ascertain the optimal embedding dimension E* required for SSR (which may not necessarily match the true dimension of the original dynamical system). Its fundamental concept lies in the notion that even for a chaotic time series, future values can be predicted using similar past values[1]. When applied in the state space, this process entails finding several neighboring points closest to the predicted point in terms of Euclidean distance for averaging and deriving the prediction (essentially the nearest neighbor prediction within the state space). The number of neighboring points is E+1 (E+1 being the minimum number of points required to form a bounded simplex in an E-dimensional space). Subsequently, predictions are computed across different embedding dimensions E, and the dimension E* that yields predictions closest to the actual values is considered the optimal embedding dimension

**3.The S-map test**

S-map, short for Sequentially Locally Weighted Global Linear Map, essentially constitutes a locally weighted linear regression model within phase space[2]. It serves as a nonlinear extension of the standard Vector Autoregression (VAR) model, differing in that the regression coefficients (or weights) in the S-map are determined by the Euclidean distance between points on the attractor in the state space, rather than their temporal proximity. Similar to simplex projection, S-map is also a method for prediction within the state space. However, unlike simplex projection, S-map doesn't solely rely on neighboring points around the prediction object; instead, it utilizes the entire dataset with specific weighted values for prediction[3,4].

**Reference**

1. Sugihara G, May RM. Nonlinear forecasting as a way of distinguishing chaos from measurement error in time series. Nature. 1990;344: 734–741. doi:10.1038/344734a0

2. Sugihara G, Grenfell BT, May RM, Tong H. Nonlinear forecasting for the classification of natural time series. Philosophical Transactions of the Royal Society of London: Mathematical, Physical and Engineering Sciences. 1994;348: 477–495. doi:doi:10.1098/rsta.1994.0106

3. Cenci S, Sugihara G, Saavedra S. Regularized S-map for inference and forecasting with noisy ecological time series. 2019;10: 650–660. doi:https://doi.org/10.1111/2041-210X.13150

4. Deyle ER, May RM, Munch SB, Sugihara G. Tracking and forecasting ecosystem interactions in real time. P Roy Soc B-Biol Sci. 2016;283. doi:ARTN 20152258 10.1098/rspb.2015.2258
