# Supplementary figures and images for: Using empirical dynamic modeling to identify the impact of meteorological factors on hemorrhagic fever with renal syndrome in Weifang, Northeastern China, from 2011 to 2020
Source: PLoS Negl Trop Dis. 2024 Jun 6;18(6):e0012151. doi: 10.1371/journal.pntd.0012151 (PMC11185475; doi:10.1371/journal.pntd.0012151)

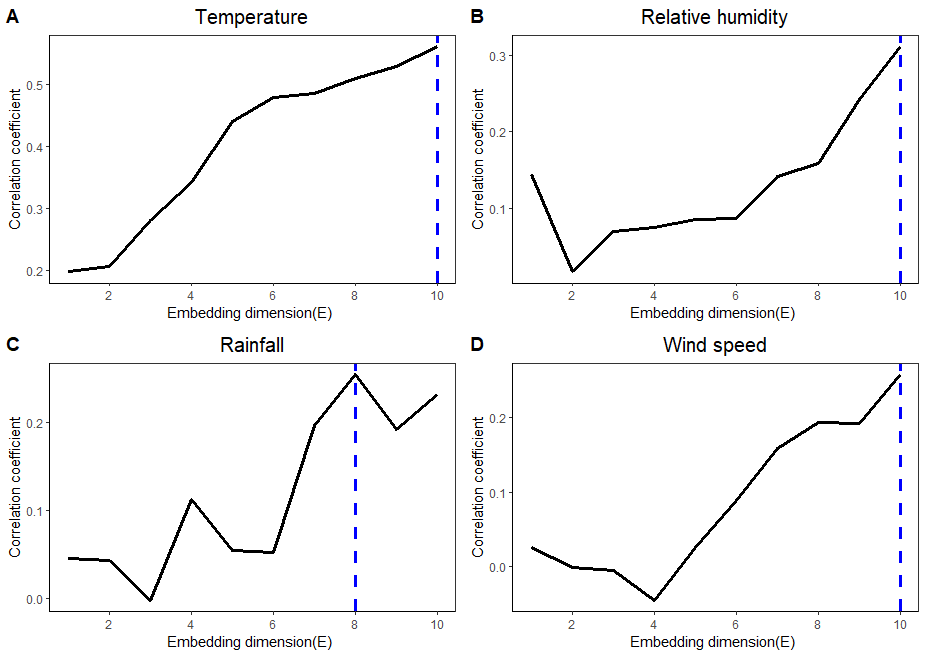

Supplement: S1 Fig — The output is a data frame with columns E and rho detailing the embedding dimension and Pearson correlation coefficient between the simplex projected forecast at Tp = 1 timestep ahead, and the observed data over the pred indices. The optimal embedding dimensions for Temperature(A), Relative humidity(B), Rainfall(C), and Wind speed(D) obtained here for further analyses are 10,10,8, and 10, respectively. (TIF) [file pntd.0012151.s002.tif]

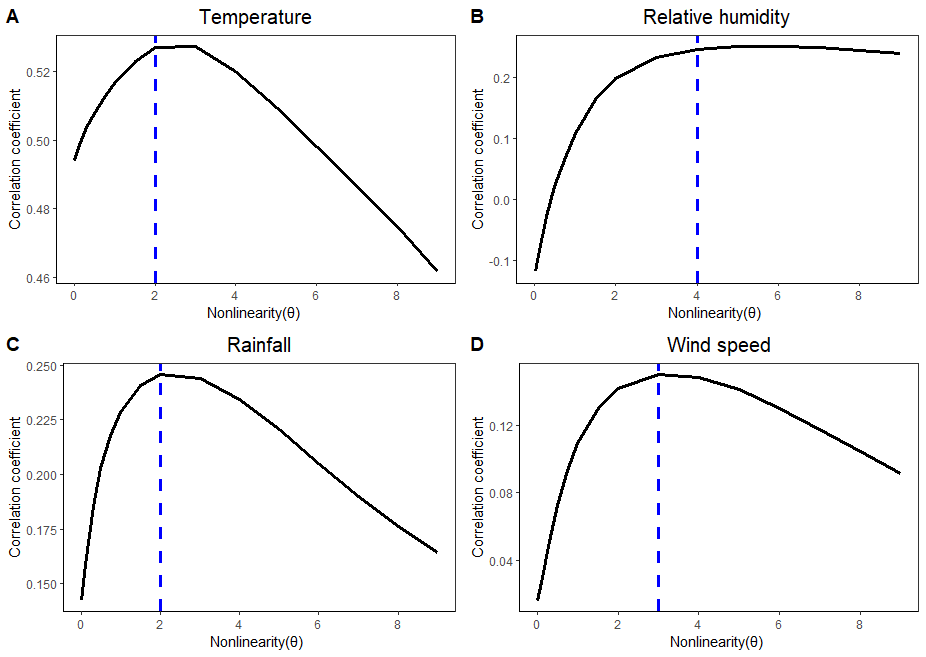

Supplement: S2 Fig — The S-map test for nonlinearity confirms that all variables have nonlinear state dependence. If the optimal θ>0, the forecast given by the S-map depends on the local state of the predicted points, then the system is state-dependent. If θ = 0 then all points are weighted equally, and the system is linear. By comparing the performance of equivalent linear (θ = 0) and nonlinear (θ>0) S-map models, one can distinguish nonlinear dynamical systems from linear stochastic systems. The optimal θ of Temperature(A), Relative humidity(B), Rainfall(C), and Wind speed(D) are all greater than 0, which means the systems of these variables are all nonlinear and thus motivate the use of EDM. (TIF) [file pntd.0012151.s003.tif]

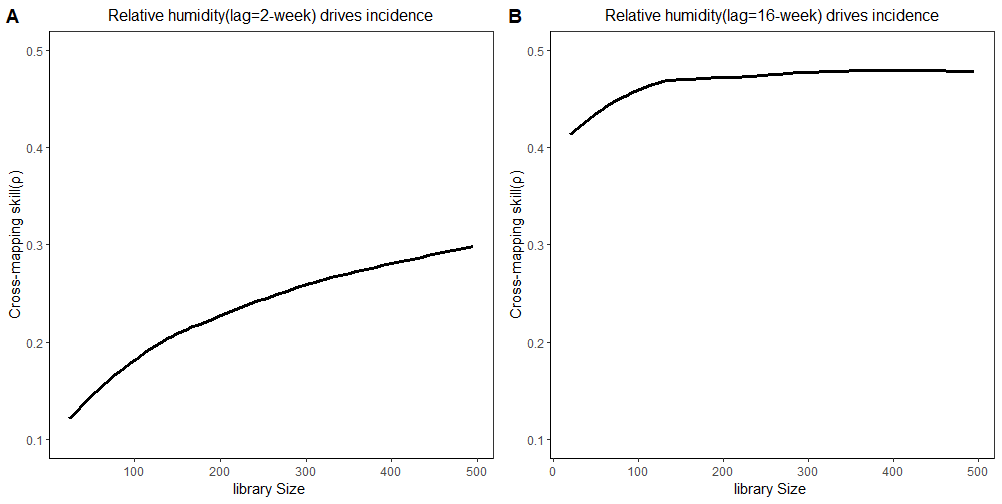

Supplement: S3 Fig — The cross-mapping skill ρ between relative humidity with a lag of 2 weeks and the HFRS incidence did not converge as library size(the sample size) increased (A). The cross-mapping skill ρ between relative humidity with a lag of 16 weeks and the HFRS incidence increases with the library size and finally converges to 0.48 (B). Relative humidity with a lag of 16 weeks can drive the incidence of HFRS. (TIF) [file pntd.0012151.s004.tif]

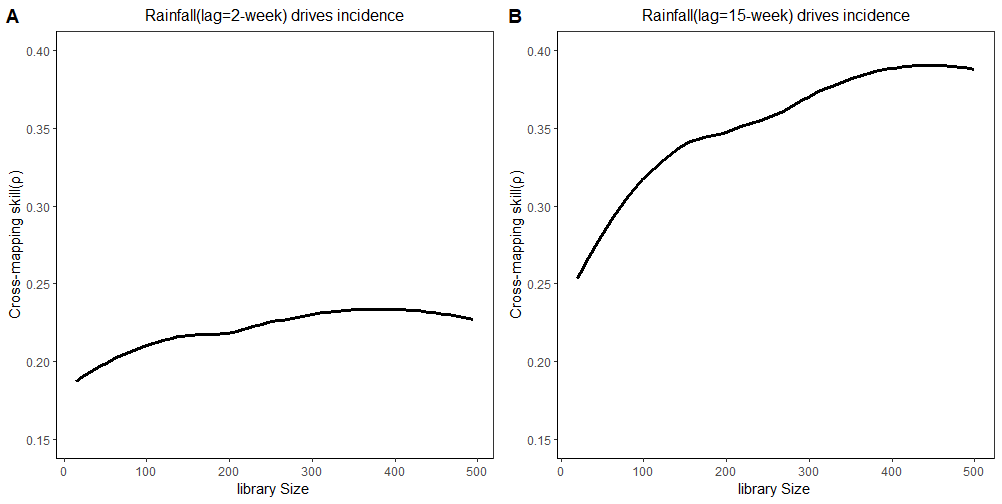

Supplement: S4 Fig — The cross-mapping skill ρ between rainfall with a lag of 2 weeks and the HFRS incidence did not converge as library size(the sample size) increased and began to decline when the library size exceeded 400 (A). The cross-mapping skill ρ between rainfall with a lag of 15 weeks and the HFRS incidence increases with the library size and finally converges to 0.38 (B). Total rainfall with a lag of 15 weeks can drive the incidence of HFRS. (TIF) [file pntd.0012151.s005.tif]

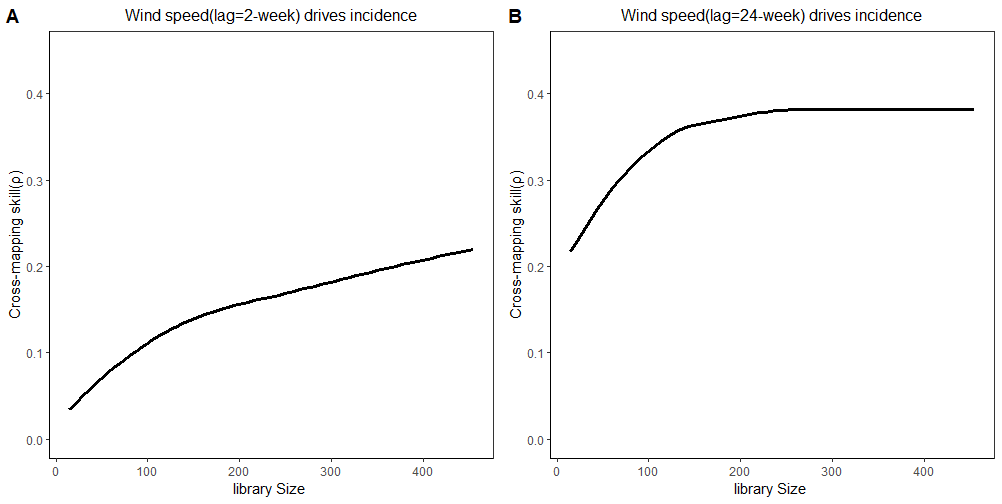

Supplement: S5 Fig — The cross-mapping skill ρ between wind speed with a lag of 2 weeks and the HFRS incidence did not converge as library size(the sample size) increased (A). The cross-mapping skill ρ between wind speed with a lag of 24 weeks and the HFRS incidence increases with the library size and finally converges to 0.39 (B). Wind speed with a lag of 24 weeks can drive the incidence of HFRS. (TIF) [file pntd.0012151.s006.tif]
